# Solitons in PT-symmetric periodic systems with the logarithmically saturable nonlinearity

**DOI:** 10.1038/srep32990

**Published:** 2016-09-06

**Authors:** Kaiyun Zhan, Hao Tian, Xin Li, Xianfeng Xu, Zhiyong Jiao, Yulei Jia

**Affiliations:** 1 College of Science, China University of Petroleum, Qingdao 266580, China; 2 Department of Physics, Harbin Institute of Technology, Harbin 150001, China; 3 College of Pipeline and civil engineering, China University of Petroleum, Qingdao 266580, China

## Abstract

We report on the formation and stability of induced solitons in parity-time (PT) symmetric periodic systems with the logarithmically saturable nonlinearity. Both on-site and off-site lattice solitons exist for the self-focusing nonlinearity. The most intriguing result is that the above solitons can also be realized inside the several higher-order bands of the band structure, due to the change of nonlinear type with the soliton power. Stability analysis shows that on-site solitons are linearly stably, and off-site solitons are unstable in their existence domain.

The optical propagation dynamics in nonlinear periodic systems has become a considerate topic in nonlinear optics, because of their intriguing physical properties and their potential applications^1^. One of the physical mechanisms that support the existence of spatial solitons is the optical induced lattices, these periodic structures with or without defects induced by nonlinearity can confine a light beam propagating in their interior or along the surface, many fantastic optical solitons supported by photonic and optical lattices have been investigated theoretically^2^ and observed experimentally^3^. Since their existence was predicted by Musslimani *et al*. in ref. 4, optical solitons in parity-time (PT) symmetric periodic potentials have attracted much special interest for their unique properties. Noteworthily, a new integrable nonlocal nonlinear Schrödinger equation with PT-symmetry is introduced by Ablowitz *et al*., and it is shown that pure soliton solutions can be supported by this integrable Hamiltonian system^5^. Quantum mechanics shows that a wide class of non-Hermitian Hamiltonians can also have an entirely real spectrum in a certain region of parameters provided they respect PT-symmetry, which indicates that the real part of the complex potential should be an even function of position and the imaginary part should be an odd one. It is noteworthy to point out that this condition is just necessary but not sufficient. The realization of complex PT-symmetric potentials within the framework of optics has paved the way towards several novel optical control schemes with intriguing and unexpected properties^6^. At present, spatial solitons have been studied in several types of PT-symmetric optical systems, including Kerr media^4^,^7^, nonlocal media^8^, quadratic nonlinear media^9^, superlattice^10^, mixed linear-nonlinear lattices^11^, competing nonlinear media^12^.

Theory and experiment have demonstrated that a saturable nonlinear medium is promising for the propagation and control of spatial solitons. Among these optical solitons, photorefractive optical solitons forms a specific branch^13^, thanks to their feature of formation at low laser power level, stability in more than one transverse dimension due to the saturable nonlinearity and a variety of potential important applications in all optical switching and signal processing. Another saturable mechanism that can support the existence of solitons is logarithmically nonlinearity. Although the real materials with logarithmically nonlinearity has not been realized, some meaningful physical interpretations have been given to the presence of the logarithmic nonlinearity, and it has proved useful for the modeling within the context of several nonlinear phenomena, which appears, for example, in Klein-Gordon model^14^, in atomic physics^15^,^16^, in magma transport^17^, and in optics^18^,^19^,^20^,^21^,^22^,^23^,^24^. For example, the Schrödinger equation with nonlinearity 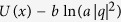
has been applied to atomic physics^15^,^16^. Here, *b* is a positive constant, *a* is a length and its value is not physically significant, since a change in its value can be compensated by multiplying the wave function by a phase, or by adding a constant potential to *U*. It is shown that the form of this logarithmic nonlinearity satisfies the condition of the separability of noninteracting subsystems, and a neutron interferometer experiment was proposed to test the physical reality of such a nonlinearity^15^,^16^. In the area of nonlinear optics, it is also difficult to find a optical medium whose optical response can satisfy this nonlinear requirement, however, the logarithmic Schrödinger equation, where the perturbed index nonlinear change for the incident beam intensity *I* is scales with ln(*I*), has been employed as a relevant equation of nonlinear wave mechanics including Gausson, periodic and quasiperiodic evolution^18^,^19^, soliton interaction^20^, modulated nonlinear solitons^21^, mighty morphing spatial solitons^22^, incoherent solitons^23^, as well as, localized soliton solutions supported by modulated lattice^24^.

In this paper, we address the existence and stability of solitons in PT-symmetric periodic systems with a saturable nonlinearity, the perturbed index nonlinear change of which varies logarithmically incident optical beam normalized intensity *I* and is scales with ln(*σ* + *I*), where *σ* = 1 and 0 is an relevant saturable parameter, corresponding to the self-focusing nonlinearity and changing nonlinearity (i.e., the logarithmic function is positive at *I* > 1 and negative at *I* < 1), respectively. Note that a logarithmic nonlinearity of this sort given by ln(*I*) was employed by Snyder and Mitchell in their study of mighty morphing spatial solitons and bullets^22^. In their model, there is a concern only for the tails of the beam at an infinite distance from its center. If *I* ≫1, negligible power exists in the tails for intensities below *I* < 1. In this model *σ* = 0, the limit *I* → 0 might appear unphysical for the realistic materials^22^. In fact, the Schrödinger equation with nonlinearity ln(*I*) can support stable and localized Gaussian soliton solutions of the form 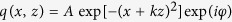, where *A* is the soliton amplitude, *k* and *φ* represent the soliton frequency and phase, respectively^19^. In this paper, we investigate the presence of stable soliton solutions of the nonlinear Schrödinger (NLS) equation with the above two logarithmically nonlinearity, which can provide valuable insight into other nonlinear wave mechanics and still maintain the characteristic features of the underlying physical process. For both mentioned types of nonlinearities, there exist two distinct types of solitons, namely on-site and off-site solitons, depending on whether the soliton is centered at a maximum or minimum of the real part of PT-symmetric periodic potential. The properties of these solitons with self-focusing nonlinearity (σ = 1) are qualitatively similar to those of solitons in nonlinear Kerr media supported by PT-symmetric potentials. The most intriguing result is that the above solitons in the case of σ = 0, can be realized inside the several higher-order bands of the band structure, due to the change of nonlinear type with the soliton power. The stabilities of these solitons are also investigated.

## Results

### Theory model

**Figure 1:**
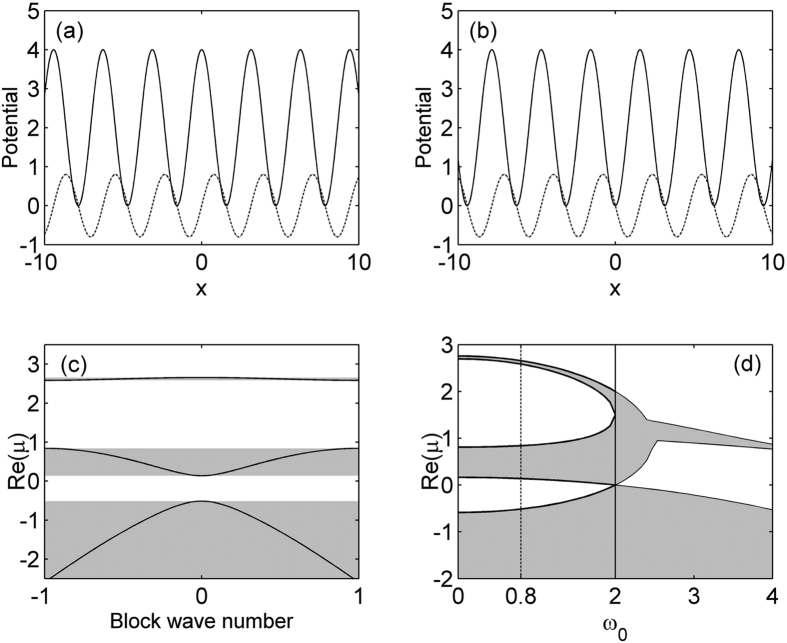
PT-symmetric potential for *V*_0_ = 4, *ω*_0_ = 0.8 and (**a**) *φ* = 0, (**b**) *φ* = *π*/2(the solid curves represent the real part of the potential, whereas the dotted curves represent the imaginary part of the potential). (**c**) The band gap structure corresponding to the lattice profiles shown in (**a,b**). (**d**) Band gap structure with *V*_0_ = 4 for different *ω*_0_. The shaded region is the Bloch bands.

We consider the propagation of an optical beam in a PT-symmetric lattice with logarithmically saturable nonlinearity. The propagation dynamic of the beam is governed by the following one-dimensional nonlinear Schrödinger (NLS) equation for the dimensionless complex amplitude of the light field *q* :


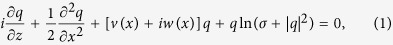


where the transverse *x* and longitudinal *z* coordinates are scaled to the characteristic beam width and diffraction length, respectively. In Eq. (1), we consider that the PT-symmetric periodic potential is given by the functions 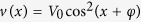 and 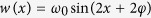, where *V*_0_ and *ω*_0_ are amplitudes of real and imaginary parts of periodic potential, respectively; *φ* = 0, *π*/2 correspond to two different complex refractive index distributions, namely on-site and off-site nonlinearity, depending on whether the real part of modes is symmetric and antisymmetric, in *x*. Note that there is no difference between the above two refractive index landscapes except a *π* phase shift, as shown in Fig. 1(a,b). In this paper, we just study the soliton solutions on both sides of the lattice center (*x* = 0), in fact, both on-site (odd mode) and off-site (even mode) solutions exist for the two values of *φ* at appropriate initial conditions. Figure 1 (a,b) show the profiles of the PT-symmetric periodic potential for *V*_0_ = 4 and *ω*_0_ = 0.8 with *φ* = 0 and *π*/2, respectively. *σ* is an relevant saturable parameter. According to the values of *σ* one can distinguish two different cases. (i) *σ* = 1: it represents a self-defocusing nonlinearity; (ii) *σ* = 0: the logarithmic function is positive at |*q*|^2^ > 1 and negative at |*q*|^2^ < 1; that is to say, the type of nonlinearity would be changed with light intensity.

We search the stationary soliton solutions of Eq. (1) in the form *q* = *f*(*x*)exp(*iμ*z) that can be characterized by the propagation constant *μ*, *f*(*x*) = *h*(*x*) + *ie*(*x*) is a complex-valued function. Eq. (1) can be rewritten in the following coupled equations,


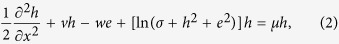



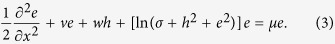


The stationary solutions can be solved numerically by the Newton’s iteration, spectral renormalization^25^ and developed squared-operator methods^26^. The total power of the system can be defined as: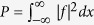. Furthermore, in order to comprehensively examine the soliton linear stability, we search for perturbed solution to Eq. (1) in the form^27^:


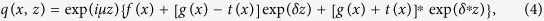


where *g*(*x*) and *t*(*x*) are small perturbations that can grow with the complex rate *δ* = *δ*_r_ + *iδ*_i_ upon propagation and the asterisk means complex conjugation. Linearization of Eq. (1) around *f*(*x*) leads to the following eigenvalue problem,


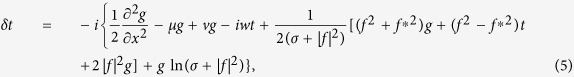



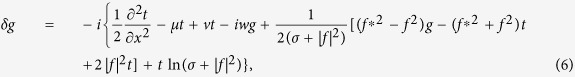


which we solve numerically to find the growth rate. If *δ*_r_>0, solitons are unstable, otherwise, they are stable.

It is instructive to analyze the linear propagation of light beams in the optical lattice. When the energy flows of soliton beams are small, the optical lattices induce a bandgap structure in the linear Schrödinger spectral problem, 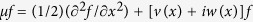. Bounded solutions of this linear equation are called Bloch modes, and the corresponding frequencies *μ* form the Bloch bands. Based on the Floquet-Bloch theory, we can obtain the Bloch spectrum by solving the above equation with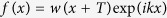, where *k* is the Bloch wave number bounded between −1 ≤*k* ≤ 1, *w*(*x*) = *w*(*x* + *T*) is a Bloch function with the same periodicity as the lattices. A typical lattice spectrum obtained by the plane wave expansion method is depicted in Fig. 1(c) at *V*_0_ = 4 and *ω*_0_ = 0.8. All possible propagation constant values are arranged into bands (gray regions), where Eq. (1) admits Bloch wave solutions, while in the gaps (white regions) periodic waves do not exist. These gaps from top to bottom are called sequentially as the semi-infinite band gap (*μ* ≥ 2.657), first finite band gap (2.584 ≥ *μ* ≥ 0.8407), second finite band gap (0.138 ≥ *μ* ≥ −0.5137), and so on in this paper. It should be pointed out that there exists a phase transition point (or threshold) 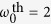, below which all the propagation eigenvalues are real. Once *ω*_0_ exceeds this critical value, an abrupt phase transition occurs because of the spontaneous symmetry breaking, the first two bands start to merge together and form an oval-like structure and a complex band diagram forms, as shown in Fig. 1(d). Further increasing *ω*_0_, it is shown that the real parts of the first two bands begin to overlap with each other, with the imaginary parts taking nonzero opposite signs.

### Lattice solitons in PT-symmetric periodic systems with logarithmical nonlinearity

To begin with, in this section, we investigate solitons and their stability supported by PT-symmetric periodic lattice with logarithmical self-focusing nonlinearity (*σ* = 1). Numerically, we find two types of lattice solitons bifurcating from band edge in the semi-infinite band gap.

**Figure 2:**
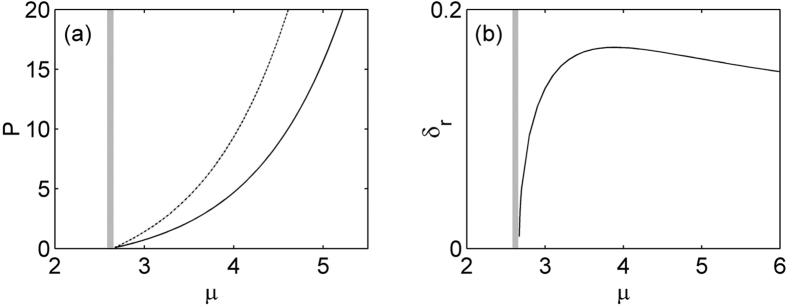
(**a**) Power of on-site (solid line) and off-site (dashed line) lattice solitons versus propagation constant for *σ* = 1. (**b**) Unstable growth rate *δ*_r_ versus propagation constant for off-site lattice solitons. For all case *V*_0_ = 4 and *ω*_0_ = 0.8.

**Figure 3:**
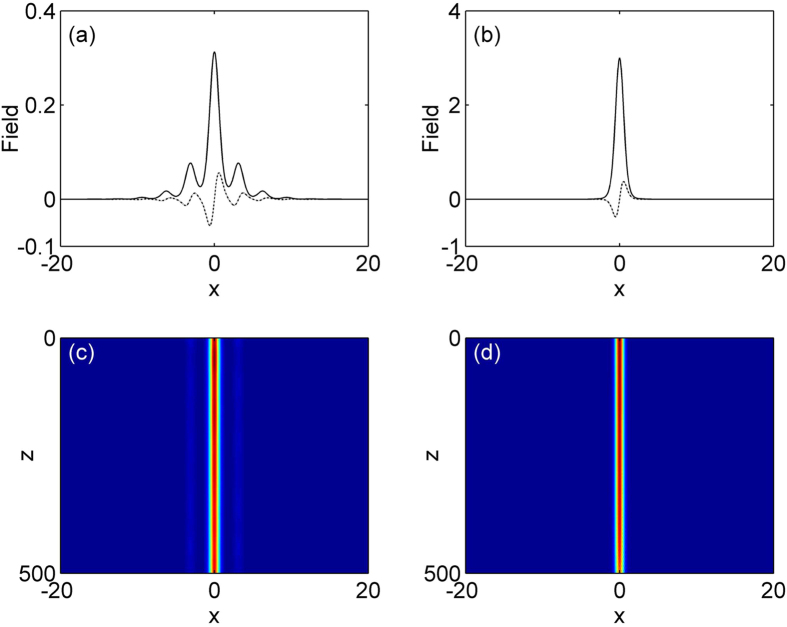
Examples of on-site lattice soliton profile (the solid curves show the real parts and the dotted corves show the corresponding imaginary parts) at (**a**) *μ* = 2.75 and (**b**) *μ* = 4.5. (**c,d**) Stable propagations of soliton shown in (**a,b**).

In the nonlinear case, Eq. (1) support a family of on-site lattice soliton at *φ* = 0 and *σ* = 1 and the results are shown in Figs 2 and 3. Figure 2(a) gives the existence curves (solid line). Obviously, this family of solution bifurcates from the base of the first band into the semi-infinite band gap. *P* is a monotonic function of *μ*, and increases with *μ* in the existence domain. Representative profiles of two solitons at low and high powers are shown in Fig. 3(a,b). Notice that real part of on-site lattice soliton modes are centered at a refractive index maximum, and as the total power increases, these solitons have the tendency to become more spatially localized inside the lattice. A comprehensive linear instability analysis based on Eqs. (5) and (6) is performed. Our result shows this family of lattice solitons in the semi-infinite band gap is completely stable in the entire existence domain. We also test the stability of the solitons by direct simulation of propagation of perturbed soliton solutions in Eq. (1). Figure 2(c,d) show two examples of the stable evolution of these solitons corresponding to profiles displayed by Fig. 2(a,b) at *μ* = 2.75 and 4.5, which are robustness and propagate without any noticeable deformations under 5% random initial perturbation.

**Figure 4:**
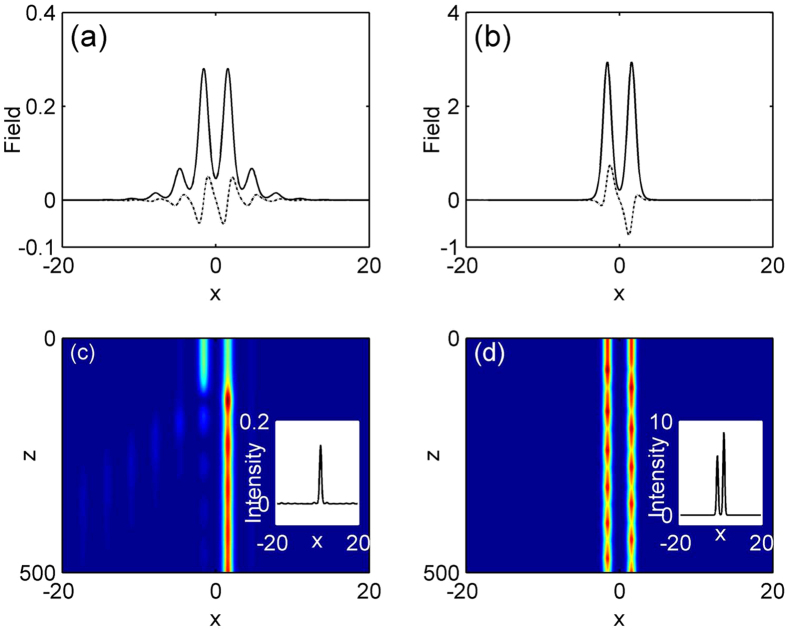
Examples of off-site lattice soliton profile at (**a**) *μ* = 2.75 and (**b**) *μ* = 4.5. (**c,d**) Unstable propagations of soliton shown in (**a,b**). The insets show soliton intensity patterns after a distance of z = 500.

We also find that a family of off-site lattice soliton supported by PT-symmetric periodic lattice with logarithmical nonlinearity can exist in the semi-infinite band gap at *φ* = *π*/2 and *σ* = 1. Corresponding real parts of soliton mode are centered at a refractive index minimum. Figure 2(a) shows the existence domain of such off-site lattice solitons (dash lined). Apparently, for the existence curve of off-site lattice soliton, a similar trend is observed to on-site lattice solitons, the soliton energy flow monotonically increases with the increase of propagation constant *μ*. As can be seen in Fig. 2(a), the power of off-site lattice soliton is higher than that of off-site one at the same propagation constant. Thus the on-site soliton is energetically favorable. Two representative profiles at high and low powers are displayed in Fig. 4(a,b). Similar to the above on-site lattice solitons, these off-site solitons here also localize at high power. Linear stability analysis indicates that such off-site lattice solitons are unstable in the entire domain of their existence, where the real part of the perturbation growth rate *δ*_r_ > 0 and the exponential instability develops, shown in Fig. 2(b). These results are confirmed by direct simulations of propagation of perturbed soliton solutions in Eq. (1). Figure 4(c,d) display examples of the evolution of unstable solitons corresponding to Fig. 4(a,b). The insets in the Fig. 4 (c,d) show the intensity profiles of off-site lattice soliton after a distance of *z* = 500. When the off-site soliton has high power, under the random initial perturbation, its energy periodically shift between two adjacent lattice sites, and always exhibits chain-type evolution due to the oscillator instability. However, the soliton with low power will shift all its energy into one lattice site and evolve into an on-site lattice soliton after a distance, as shown in Fig. 4(c).

### Inband solitons in PT-symmetric periodic systems with logarithmical nonlinearity

In this section, we constrain our studies on solitons and their stability supported by PT-symmetric periodic lattice in the case *σ* = 0, where the type of nonlinearity would be changed with increasing soliton power. Both on-site and off-site inband solitons inside several higher-order bands of the linear spectrum are found.

**Figure 5:**
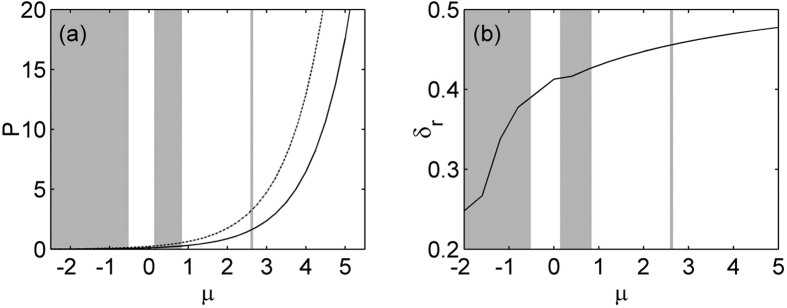
(**a**) Power versus propagation constant for on-site (solid line) and off-site (dashed line) inband solitons at *σ* = 0. (**b**) Unstable growth rate *δ*_r_ versus propagation constant for off-site inband solitons. For all case *V*_0_ = 4 and *ω*_0_ = 0.8.

**Figure 6:**
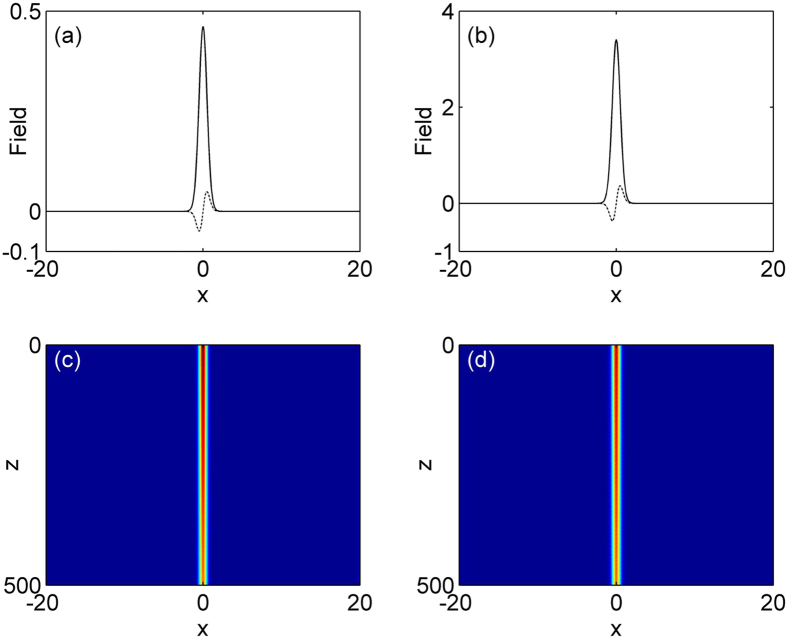
Two typical on-site inband soliton profiles at parameter (**a**) *μ* = 0.5 and (**b**) *μ* = 4.5. (**c,d**) Evolutions of on-site inband soliton under random initial perturbation corresponding to (**a,b**), respectively.

In the model with *φ* = 0 and *σ* = 0, the existence area of the on-site inband solitons inside higher-order bands on the (*μ*, *P*) plane is presented in Fig. 5(a), which shows that the solitons with high power can be readily found in semi-infinte gap, as the power decreases, the existence curve penetrates inside the band, and further into higher-order band gap, and these solitons in the higher-order band and band gap have a very low intensity. This property is attributed to the change of nonlinear type with beam intensity. It is well known that periodic waveguide structures, including waveguide arrays, optically induced lattices, usually support Floquet–Bloch modes, all possible periodical guided modes are arranged into bands which are separated by gaps between them, and periodic waves do not exist in the gaps. Recently, it is shown that some soliton modes can reside inside the Bloch bands in some periodic systems. For example, localized solutions known as embedded solitons can exist inside the linear spectrum for discrete values of the propagation constant, which is possible if the spectrum of the linearized system possesses two branches, one corresponding to exponentially localized solutions and the other to radiation modes^28^. The another self-localized solutions, named nonlocal lattice solitons supported by thermal nonlocal nonlinearity with an infinite range of nonlocality, also can exist inside the bands of the band structure, which is attributed to the infinite range of nonlocality as well as to the boundary condition applied to the system^29^. In our models, on-site inband solitons supported by PT-symmetric periodic lattice can also be realized inside the several higher-order bands of the band structure, due to the change of nonlinear type with the soliton power. Similar to the gap solitons in other periodic systems, when the power is high (|*q*|^2^>1), Eq. (1) represents a self-focusing nonlinearity, and solitons can exist in the semi-infinite gap. Nonlinearity will be turned into self-defocusing type at |*q*|^2^<1, and solitons can be found in finite band gaps. As a result, these solitons can exist in several bands and gaps, due to the change of nonlinear type with the soliton power. It should be note, for |*q*|^2^ → 0, Eq. (1) would have an infinite nonlinearity and an unpleasant feature might appear. It requires that the light intensity should not be too low. In our simulation, it is found that the solitons with low power *P* = 0.01 can also be readily found. Figure 6(a,b) display two representative soliton profiles with low and high power at *μ* = 0.5 and 4.5, respectively. Different from lattice solitons in the previous section, these inband solitons here do not localize at high power. The stability of these solitons is tested by direct simulation and linear stability analysis. It is shown that, when these inband on-site soliton do not have extremely low powers, they are found to be stable in whole existence domain. Figure 6(c,d) show the examples of soliton stable evolution propagation corresponding to Fig. 6(a,b), which show that these inband solitons supported by the combined effect of PT-symmetric potential and logarithmic nonlinearity can propagate without any noticeable deformations under 5% random initial perturbation after a distance of z = 500.

**Figure 7:**
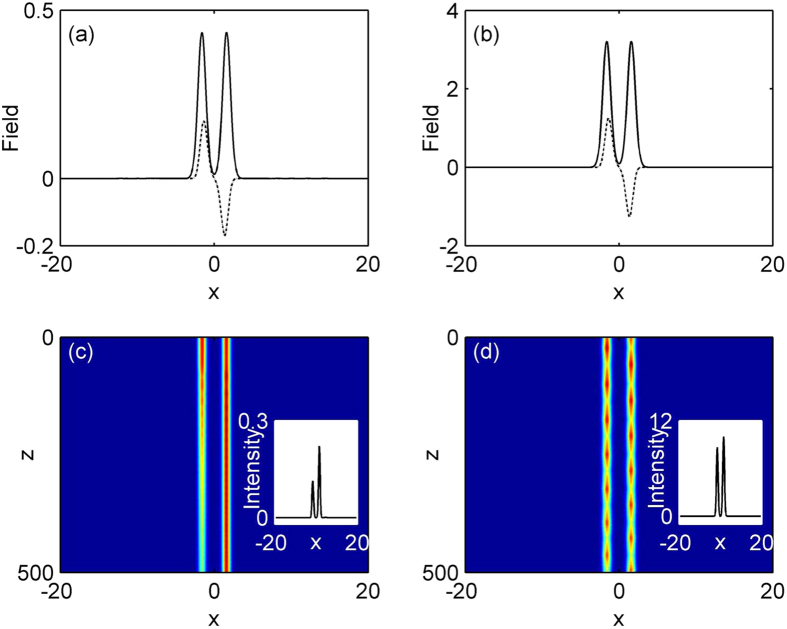
Two typical off-site inband soliton profiles at parameter (**a**) *μ* = 0.5 and (**b**) *μ* = 4.5. (**c,d**) Unstable evolutions of off-site inband soliton under random initial perturbation corresponding to (**a,b**), respectively. The insets show soliton intensity patterns after a distance of z = 500.

In this subsection, we examine off-site inband solitons inside the higher-order band of the linear spectrum. The power diagram is shown in Fig. 5(a), which reveals that these off-site inband solitons also can exist in several bands and band gaps, and the power of off-site inband soliton has a higher power than on-site one at the same propagation constant. Both the linear stability analysis and direct numerical simulation indicate that these off-site inband solitons are linearly unstable, shown in Fig. 5(b). Figure 7(a,b) depict two typical soliton profiles at parameters *μ* = 0.5 and 4.5, whose evolution propagations are shown in Fig. 7(c,d), respectively. And the intensity patterns of off-site inband soliton after a distance of *z* = 500 are shown in the insets of Fig. 7 (c,d). Similar to on-site inband solitons, these off-site inband solitons also do not localize in high power domain. Figure 7(c) shows that low power soliton also shift its energy into one lattice site but not be found to evolve into an on-site soliton, which is different from the off-site lattice soliton at *φ* = *π*/2 and *σ* = 1, shown in Fig. 4(c). In high power domain, it also always exhibits chain-type evolution due to the oscillator instability, which is similar to the unstable evolution of off-site lattice soliton. This property is attributed to the nature of nonlinearity associated with the soliton power in equation (1). In the high soliton power domain, numerical beam propagation in this ln(*I*) model is indistinguishable from that of the ln(*σ* + *I*) nonlinearity.

### Summary and discussion

To conclude, Solitons and their stability in PT-symmetric periodic systems with the logarithmically saturable nonlinearity have been analyzed. Two families of solitons, i.e., on-site and off-site solitons supported by the PT-symmetric periodic potential can be formed for both the self-focusing nonlinearity and varied nonlinearity. Both on-site and off-site lattice solitons for the case of self-focusing nonlinearity have been found in the semi-infinite band gap, and bifurcate from the edge of Bloch band into the corresponding band gap. On-site lattice solitons belonging to the semi-infinite band gap can propagate stably in whole existence domain, but off-site lattice solitons belonging to this band gap are unstable in the entire domain of their existence. These off-site lattice solitons with high power exhibit chain-type unstable evolution under random initial perturbation, whereas an off-site soliton in low power domain would evolve into an on-site lattice soliton after a distance. In the case *σ* = 0, both the on-site and off-site inband solitons can also be realized in the PT-symmetric periodic systems, where the type of nonlinearity would be changed with light intensity. The most intriguing property is that the two families of solitons can exist inside the high-order bands of the band structure. This property is attributed to the change of nonlinear type with the soliton power. It is shown that existence curves of inband solitons penetrate inside the relatively high-order band gap, where solitons have very low power. Similar to lattice solitons under a self-focusing nonlinearity, on-site inband solitons are linearly stably, and off-site inband solitons are unstable in their existence domain. At high power domain, it also always exhibits chain-type evolution, the low power soliton can also shift its energy into one lattice site but not be found to evolve into an on-site soliton.

A critic might question whether our model can be realized physically, indeed, the logarithmic Schrödinger equation has no direct application in nonlinear optics. This law arises in various fields of contemporary physics. Various meaningful physical interpretations have been given to the presence of the logarithmic potential^30^. Our results about the beam propagations in the logarithmically nonlinear systems might provide some valuable insight into other nonlinear wave mechanics. These results can be extended to other periodic settings, where the type of nonlinearity can be changed with the intensity, and may provide several novel ways for beam controlling in micro structure. In addition, in this paper, we address the general properties of logarithmical solitons supported by PT-symmetric lattices below the phase transition points. Nonlinear wave dynamics with logarithmically nonlinearity in PT-symmetric lattices near the phase transition points and in nonlinear real lattice have not been explored yet.

## Additional Information

**How to cite this article**: Zhan, K. *et al*. Solitons in PT-symmetric periodic systems with the logarithmically saturable nonlinearity. *Sci. Rep.* **6**, 32990; doi: 10.1038/srep32990 (2016).
